# Bunions

**Published:** 1901-03

**Authors:** 


### Bunions.

Isadore Dyer (*New Orleans Medical and Surgical Journal*) advises wearing properly adjusted shoes and protecting the parts with a simple cotton and collodion dressing. Tincture of iodine and bathing the foot twice a day in starch water are also mentioned.

